# Prenatal Sex Role Stereotypes: Gendered Expectations and Perceptions of (Expectant) Parents

**DOI:** 10.1007/s10508-023-02584-9

**Published:** 2023-03-21

**Authors:** Roland Imhoff, Lisa Hoffmann

**Affiliations:** 1 Social and Legal Psychology, Johannes Gutenberg University Mainz, Binger Str. 14-16, 55122 Mainz, Germany; 2 Social and Legal Psychology, University of Bonn, Bonn, Germany

**Keywords:** Sex roles, Gender roles, Parents, Sex typing, Socialization, Birth, Sex differences

## Abstract

People assign attributes to a different degree to other persons depending on whether these are male or female (sex role stereotypes). Such stereotypes continue to exist even in countries with lower gender inequality. The present research tested the idea that parents develop sex role consistent expectations of their babies’ attributes based on fetal sex (by ultrasound diagnostic), as well as gendered perceptions of their recently newborn babies. A total of 304 dyads of predominantly White expecting parents from Germany were followed over the course of pregnancy until after the birth and completed a sex role inventory on their babies’ expected (before birth) as well as perceived traits (after birth). Specifically, they rated to what extent they expected their babies to have normatively feminine traits (e.g., soft-spoken and warm) and normatively masculine traits (e.g., independent and assertive) twice before birth (first half of pregnancy, six weeks before due date) and to what extent they perceived their baby to have these traits eight weeks after birth. The results suggested that fathers held gendered expectations and perceptions, whereas mothers did not. These results suggest that male and female babies are likely to encounter sex role stereotypes about their alleged attributes as soon as their birth.

## Introduction

Sex role stereotypes are a pervasive phenomenon. As a whole body of the literature suggests, people hold beliefs about how men allegedly are and how women allegedly are. Frequently, women are described as more caring, tender and emotional, whereas men are seen as more agentic, assertive and dominant. This is not only true for abstract stereotypical beliefs, but it is also reflected in how men and women describe themselves in self-reports. Typically, many rate themselves as higher on items reflecting either agentic traits (e.g., assertiveness), whereas women have higher self-ratings on communal traits (e.g., warm, caring, and tender-mindedness; Feingold, 1994), a pattern that has become increasingly pronounced over the past 70 years (Eagly et al., 2020).

Although many authors insist on the possibility that some of such differences may be biologically grounded, there is a large consensus that at least part of these differences come to life by a process called sex typing (Birns, 1976; Block, 1973, 1983; Maccoby & Jacklin, 1974), the transmission of sex-typical expectations from socialization agents to the child. As so eloquently put by de Beauvoir (1949, p. 267), one is not born, but rather becomes, a woman or man (“On ne naît pas femme: on le deviant”). Of these socialization agents, parents play a pivotal role and hence do their expectation and potentially differential treatment of children assigned the male or female gender at birth. In everyday life, this is, e.g., reflected in using stereotypical color schemes for the nursery, gender-specific clothing for newborns, and gender reveal parties that have been becoming increasingly popular (Gieseler, 2018). In this regard, it seems almost trivial to assume that this focus on gender and potential differences, even at such an early age, might not only produce different behaviors in parents as described above but also shape their perception, e.g., via confirmation bias (Nickerson, 1998; Oeberst & Imhoff, in press). Parent’s stereotyped perception and expectations could have an impact on the children later on (Maccoby & Jacklin, 1974; see also below).

Already for half a century, a great body of research has explored differential reactions of parents to male and female babies, but also their different perceptions of male and female babies. Parents reported different socialization practices for boys than for girl with a general pattern being that agentic competence was more encouraged in boys, but interpersonal expressive skills were fostered in girls (Block, 1973). This is even true when actual potential temperamental differences between baby boys and baby girls (Campbell & Eaton, 1999; Eaton & Enns, 1986) are excluded as an explanatory mechanism in so-called “labelling” studies. Here, babies of both sexes are neutrally clothed and either labelled as a “boy” or a “girl” whereupon adult behavior toward the baby and their impressions of the baby are recoded as dependent variable (for a review of the suggestive, but not overwhelming evidence for the effect of such labels, see Stern & Karraker, 1989). Despite somewhat inconsistent findings over a large array of (typically small-scale) studies, gendered parenting practices have been claimed to be unmistakable “if you know where to look” (Mesman & Groeneveld, 2018).

Typically, the reasoning behind why parents behave differently toward babies perceived as differing in their sex is that they act in accordance with their preconceived gendered expectations which may then be reinforced by infants behaving according to these affordances and hence confirming parents’ expectations (Maccoby & Jacklin, 1974). And, indeed, a number of studies document such gendered expectations that is different expected qualities of babies depending on their sex. In a seminal study by Rubin et al. (1974), parents of newborns rated daughters as more soft, fine-featured, awkward, and weak, but sons as more firm, big, well-coordinated and strong—despite no objective differences between the sexes in weight, length, or Apgar scores. A more recent study (Karraker et al., 1995) replicated this finding, albeit with smaller effect sizes. Such declining effect sizes may indicate changing social norms and stereotypes, pointing to the relevance of enriching the literature with new data in certain time intervals.

In labelling studies recruiting students, but also pregnant women, “boy” babies were seen as stronger and more of a problem, whereas “girls” were perceived to be more sensitive (Burnham & Harris, 1992). Mondschein et al. (2000) found that mothers had more optimistic expectations of the motoric abilities of boys than girls—in the absence of any actual differences.

These perceptions of children often fall in-line with societal ideas of what constitutes femininity on the one hand, and masculinity on the other hand. Of the four attributes that yielded a mean level difference between the ratings of baby boys and baby girls, by far the largest was that that boys were perceived as more masculine/less feminine than girls (Karraker et al., 1995). But what exactly is meant by masculine and feminine, respectively? Many have suggested that masculinity and femininity, respectively, may serve as an umbrella term to combine the prescriptive social understandings of how a man and/or a woman is expected to be (sex roles).

Bem (1974a) had proposed a measurement of socially shared norms of what is considered masculine and/or feminine in the Bem’s Sex Role Inventory (BSRI). Masculinity is defined mostly by agentic traits and behaviors like assertiveness, forcefulness, leadership qualities, and dominance. Femininity, on the contrary, is captured by communal traits and behavior like loyalty, tenderness, warmth, and loving children. These two dimensions have also been postulated as the cardinal dimensions of personality (Leary, 1957), leadership behavior (Chemers, 1997), or stereotypes (Fiske et al., 2002; but see Koch et al., 2016). They are both theoretically and empirically orthogonal (but see Imhoff & Koch, 2017). Given that the original BSRI has been published almost 50 years ago, a legitimate concern may be—despite the ubiquity of these two dimensions in psychological research—whether they still serve as sex role typical attributes almost half a century later. Do people still see the included attributes as good examples of masculine and feminine traits, respectively? Recent meta-analytic evidence (Eagly et al., 2020) suggests that on the level of self-description men and women indeed reliably differ on agentic traits (more pronounced among men) and communal traits (more pronounced among women), a pattern that is not attenuating, but becoming more pronounced over time. Likewise, cross-temporal meta-analysis on the BSRI suggest only subtle changes in the mean scores despite slight decreases in the sex differences (Donnelly & Twenge, 2017). Given the topicality of these dimensions in adults’ self-description we were interested in whether they also perceive these characteristics in their unborn fetuses (via ultrasound diagnostic) and later their few-week-old babies—that is, at times when no differences with respect to these stereotypes would actually be expected. Differences in perception would therefore indicate that it is not the actual behavior of the children that is decisive, but that a distorted, stereotyped perception is already present at a very early stage.

The current study thus replicated and expands a previous one asking mothers and fathers to attribute different sex role stereotyped characteristics (the attributes used by Rubin et al., 1974) to their child prior to ultrasound, following the ultrasound and before the birth of the child, and immediately following birth (Sweeney & Bradbard, 1988). This study found that boys were rated as more coordinated and less fine than girls even before birth (but after the ultrasound diagnostics). Two aspects of that study, however, prevent interpreting these findings as solid evidence for prenatal gendered expectations. First, there were only twelve couples with and 12 couples without ultrasound-based based knowledge of their baby’s sex, resulting in only six boys and six girls in each group, providing very low statistical power to detect the predicted effects. Second, more problematically, the differences between infant boys and girls were based on the complete sample, including the parents who had not obtained any knowledge about their child’s sex. Specifically, the authors explicitly state that there were no differences between parents who knew and parents who did not now their child’s sex (p. 400). Together, this might suggest that even parents of a baby girl who did not know that they were expecting a girl, judged their baby as finer than parents of boys (although the results are not decomposed in such detail). It is thus unclear whether these results speak to actual sex differences (in prenatal behavior) or (ultrasound-based) gendered expectations.

### Differences Between Mothers and Fathers?

An important question of prior research has been, whether such gendered expectations (and ultimately sex-typing behavior) is symmetrical between fathers and mothers. A plethora of findings suggests that it may not. Specifically, in the seminal study by Rubin et al. (1974), fathers used very stereotypic ratings. Likewise, the socialization behavior of men has been found to be more sex typing than that of mothers (Block, 1973), for instance in their buying decision for sex (a-)typical toys (Fisher–Thompson, 1990). In a review of the then available literature, Siegal (1987) went as far as suggesting that fathers’ behavior or impressions differed significantly between boys and girls, whereas hardly any differences could be found for mothers (for a similar conclusion, see Power, 1981). This parental asymmetry is further corroborated by the fact that fathers endorse more traditional sex stereotypes at least in self-reports (Blakemore & Hill, 2008; Endendijk et al., 2013; Tenenbaum & Leaper, 2002).

### The Present Research

In the present study we sought to test the idea that parents form gendered expectations as early as learning their unborn child’s sex at ultrasound diagnostic. This is theoretically interesting because the question to what extent parent’s expectation are indeed preconceived stereotypes or merely an adaptation to different behavior and affordances provided by baby girls and boys is a seemingly unresolvable challenge (Collins et al., 2000). By looking at the prenatal phase, we can more clearly isolate parents’ preconceived expectations from simply knowing their baby’s sex. The opposite direction that unborn girls and boys behave in a way that provokes different parental expectations seems radically less likely. In addition, this study provided us with an opportunity to not only test the continued existence of such sex stereotypes, but also their differential endorsement by fathers and mothers, respectively

Mothers and father completed the Bem Sex Role Inventory (Bem, 1974a) at three time points (with slightly different wording between the first two and the third): early in pregnancy before their child’s sex was known, at the end of pregnancy after having learned baby sex from ultrasound diagnostic, and shortly after birth. The first two measurements asked for gendered expectations (to what extent they wished their baby to hold the respective [sex role in-/consistent] attributes), the last one asked for gendered perception (to what extent they believed their baby to actually have the respective [sex role in-/consistent] qualities).

In order to collect the relevant variables, we capitalized on them being included in a large-scale study collecting multiple measurements from couples from early pregnancy until after birth for other primary purposes (Hoffmann et al., in press; https://osf.io/crja6 for a complete list of collected variables).

## Method

### Participants

Couples in Germany expecting a baby were recruited for a larger study on birth experiences either via flyer by OB-GYNs or midwives, in Facebook groups, or via a Facebook advertisement in exchange for monetary compensation (for details: Hoffmann et al., in press). Participating couples received extensive information about the procedure of data collections and the different time points and response format and that the goal of the study was to study psychological processes during pregnancy, birth, and childbed (without exact details on the kind of processes, as the participant information was already ten pages without going into the detail of each question) and gave their informed consent. The original study included five measurements: between 6 and twenty-sixth pregnancy week (t1 henceforth), four to six weeks before the baby’s due date (t2), within the first week after birth (not included in the current paper), roughly eight weeks after birth (t3), as well as a follow-up six months later (not included in the current paper due to too small return rate). Sample size depended on the time of measurement due to missing completions (t1: *n* = 304, t2: *n* = 290, t3: *n* = 287). The mean age for female participants at t1 was 30.3 years (*SD* = 3.98, ranging from 20 to 39), and 32.5 years (*SD* = 4.52, ranging from 23 to 53) for male participants. Participants predominantly came from a highly educated background (48% of women and 48.7% of men had a university degree) with both partners earning an average income (most frequent option and median of the net income distribution was the range from 1500€ to 2500€ for both men and women). Information on race or ethnicity was not recorded for this study.

### Measures

The study mainly focused on birth experiences and thus most variables collected related to these. For the purpose of the current paper, we only describe the variables relevant for the reported analyses.

#### Baby Sex

Routinely, pregnant women in Germany have regular ultrasound screening at their OB-GYN. The second of these screenings is typically around the twentieth week of pregnancy and also the time when baby sex is diagnosed relatively reliably. Although some parents decide not to learn the results, most do. Legally, practitioners in Germany cannot inform parents about the baby sex before the end of the twelfth week of pregnancy. At t1 we asked both parents whether they already knew their baby’s sex (as some completed this after week 20) and 85% of mothers had no knowledge. At t2, we asked mothers whether they were expecting a boy, a girl or did not know (either because they chose not to know or because the ultrasound was not unambiguous) and 91% of mothers did know and indicated either of the two sexes.

#### Gendered Expectations and Perceptions

To track potential gendered expectations and perceptions, we adapted Bem’s Sex Role Inventory (BSRI; Bem, 1974a, 1974b) in its German version (Schneider-Düker & Kohler, 1988). This scale consists of 60 attributes out of which 20 reflect feminine attributes (e.g., soft-spoken), 20 reflect masculine attributes (e.g., assertive). In addition, 20 filler trials tap into overall socially desirable attributes (e.g., reliable), with ten of them reverse-coded (e.g., inefficient). To tap into gendered expectations, we asked both parents at the first two measurements to what extent they wished their baby to have these attributes on a scale from *do not agree at all* (1) to *fully agree* (10) (“Please indicate to what extent you wish your baby to exhibit the following quality in their later life.”). The same scale was also used for gendered perceptions. Eight weeks after birth, parents were asked to indicate for each of the 60 attributes on the same scale (“Now, that I have met my baby, I think it is…”). Reliabilities for the three subscales, measurement time, and parent can be found in Table 1. A full list of items (English translations) can be found on OSF. Although we know of no research adapting this attribute list to the rating of newborns, some of the seminal findings on parental perceptions of babies (Karraker et al., 1995; Rubin et al., 1974) used individual attributes in a very similar way, some of which show semantic overlap with BSRI attributes (e.g., strong, assertive, and active).

**Table 1:** Scale reliability and descriptive statistics of BSRI scales as a function of parent sex and (expected) baby sex for all three measurement times

	*n*	Feminine traits			Masculine traits			Filler traits (social desirability)		
		*M*	SD	α	*M*	SD	α	*M*	SD	α
T1: Desired traits										
Mothers without knowledge of baby sex	259	6.63	0.69	.77	6.97	0.83	.85	8.66	0.70	.87
Mothers with knowledge of baby sex	44	6.60	0.76		6.93	0.96		8.70	0.66	
Fathers without knowledge of baby sex	262	6.55	0.74	.74	7.28	0.85	.85	8.49	0.79	.87
Fathers with knowledge of baby sex	42	6.46	0.66		7.19	0.96		8.38	0.94	
T2: Desired traits										
Mothers expecting baby girl	132	6.64	0.62		6.94	0.84		8.62	0.69	
Mothers expecting baby boy	131	6.50	0.68	.75	6.83	0.78	.84	8.62	0.65	.86
Mother without knowledge of baby sex	27	6.50	0.77		6.48	1.00		8.24	0.93	
Fathers expecting baby girl	132	6.60	0.75		7.11	0.93		8.26	0.90	
Fathers expecting baby boy	129	6.48	0.75	.77	7.30	0.89	.86	8.42	0.74	.88
Fathers without knowledge of baby sex	27	6.47	0.60		6.98	0.54		8.26	0.69	
T3: Perceived traits										
Mothers to newborn girl	141	6.83	0.88	.82	6.86	1.05	.89	7.75	1.07	.90
Mothers to newborn boy	146	6.55	0.82		6.69	0.99		7.67	1.05	
Fathers to newborn girl	141	6.68	0.97	.86	6.91	1.11	.91	7.53	1.25	.93
Fathers to newborn boy	145	6.44	0.90		7.00	1.10		7.47	1.18	

All variables on a scale from 0 to 10

#### Parental Self-Ratings

To allow an estimation whether the BSRI does capture meaningful differences as a function of sex, parents also indicated at each measurement time for each attribute of the BSRI whether they wished to hold this attribute themselves. This served as a validation of the BSRI scales if they produced predictable differences between fathers (more masculine, less feminine traits) and mothers. Initially, we had also planned to explore whether a baby’s sex would make parents assimilate their self-ratings into the direction of the baby’s sex (e.g., fathers wishing to be soft and considerate toward girls more so than boys). As these results did not provide any support for such effect, we do not report them here for brevity.

#### Baby’s Temperament

To control for actual baby temperament, we recorded the Ecological Momentary Assessment (EMA; Stone & Shiffman, 1994) after birth. EMA uses repeated sampling on current (emotional) states and behaviors in the participant’s natural environment (Shiffman et al., 2008). We conducted a time-based sampling, thus, at a random time of the day, participants perceived a link to our online questionnaire on their mobile phones. The link was sent daily in the first 14 days after birth. In the following four weeks (week 3 to 6), this frequency was reduced to once per week (maximally 19 measurements in total). The questionnaire included items on (emotional) well-being, breastfeeding, wound healing, and the baby’s well-being and behavior (see Hoffmann et al., in press). Only the latter is relevant for the present study. The baby’s well-being and behavior included overall ten items; the answer format was a semantic differential. The following four items were included in the analyses for the assessment of the baby’s temperament: screamed a lot to barely screamed, very calm to not calm at all, very exhausting to not exhausting at all, easy to comfort to difficult to comfort. For each item, we estimated reliability by averaging separately for odd and even measurement times and estimated per item reliability (*αs* ≥ 0.84 for mothers; *αs* ≥ 0.78 for fathers). The aggregated estimates per item and parent were then subjected to exploratory factor analyses with both analyses clearly suggesting a one-factorial solution. We thus recoded two items and computed composite scores of temperament (*α* = 0.94 for mothers; *α* = 0.94 for fathers). High scores indicated that the baby was overall not exhausting, barely screaming, easy to comfort, and very calm.

### Data Analysis

We screened all core variables for their distribution and found no severely skewed scales. Only complete cases entered the data analysis at the respective time points, but there were very few missing data (although some couples terminated participation, but their number is too small to warrant meaningful analyses regarding confounding variables). For the combined analysis of mothers and fathers across *t*1 and *t*2, data were deleted listwise. Thus, any dyad with any missing data was excluded from analyses. As all relevant variables were entered on Likert scales and hence restricted by these scales, we did not screen for outliers.

## Results

### Are the BSRI Attributes Still Timely for Our Sample?

**Fig. 1:**
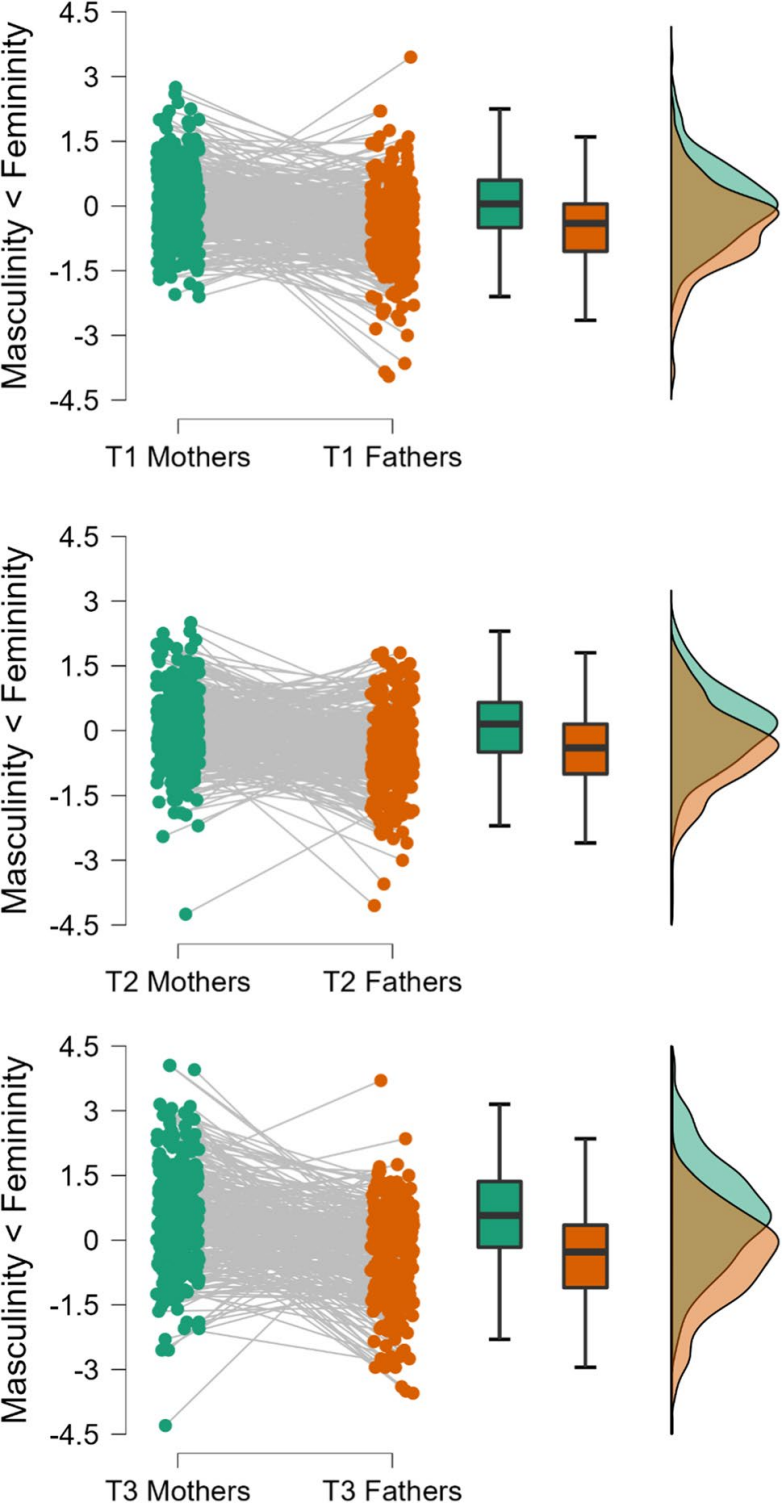
Raincloud plots of ideal self-images of mothers on fathers on BSRI femininity and masculinity scales (differences score displayed) at all three measurement waves

Although sex roles are not expected to map perfectly on self-identified sex, the very idea of sex roles is indeed that they are perceived as sufficiently prescriptive to find that women describe themselves more in feminine traits and men more in masculine traits. As elaborated above, several authors have questioned whether the attributes identified as masculine and feminine, respectively, in the 1970s are still timely descriptors of current sex roles. We thus tested whether fathers and mothers differed in their self-ascribed masculinity and femininity, respectively. For all analyses involving the BSRI (see also below) we calculated difference scores of the endorsement feminine over masculine traits in order to ease interpretation. Mathematically, an interaction of parent role (mother vs. father) and BSRI scale (masculinity vs. femininity) thus translates into a simple mean difference on the difference score. We conducted paired t test within each couple at all three measurement points. As expected, mothers perceived themselves to be more feminine than fathers did at T1, *t*(302) = 7.84, *p* < 0.001, *d*_*z*_ = 0.45, at T2, *t*(287) = 7.67, *p* < 0.001, *d*_*z*_ = 0.45, as well as at T3, *t*(289) = 9.78, *p* < 0.001, *d*_*z*_ = 0.57 (Fig. 1). Although an inspection of the descriptive data shows substantial overlap between mothers and fathers, in light of the robust pattern and healthy effect size despite being developed almost 50 years ago, it seemed useful to proceed with the BSRI to tap into gendered expectations and perceptions.

### Do Parents Desire Their Babies to Have More Feminine/Masculine Traits Based on Ultrasound Diagnostic of Sex?

We tapped into parents’ desired traits for their children at two time points. These were chosen so that most parents would not know their child’s sex at T1, but would (based on ultrasound diagnostic) at T2. A gendered expectation would be reflected in an assimilation of one’s desired traits toward the prescribed sex role of one’s baby’s sex (i.e., desiring more masculine traits for sons, more feminine ones for daughters). In order to test this, we subjected the same BSRI difference scores (femininity over masculinity) to a repeated measures ANOVA with the within-couple factors parent role (mother vs. father) and measurement time (T1 vs. T2) and the between-couple factor of presumed baby sex based on ultrasound. We excluded parents who knew their child’s sex already at T1 and those who had no ultrasound-based information on their baby’s sex at T2 (44 mothers and 42 fathers).

**Fig. 2:**
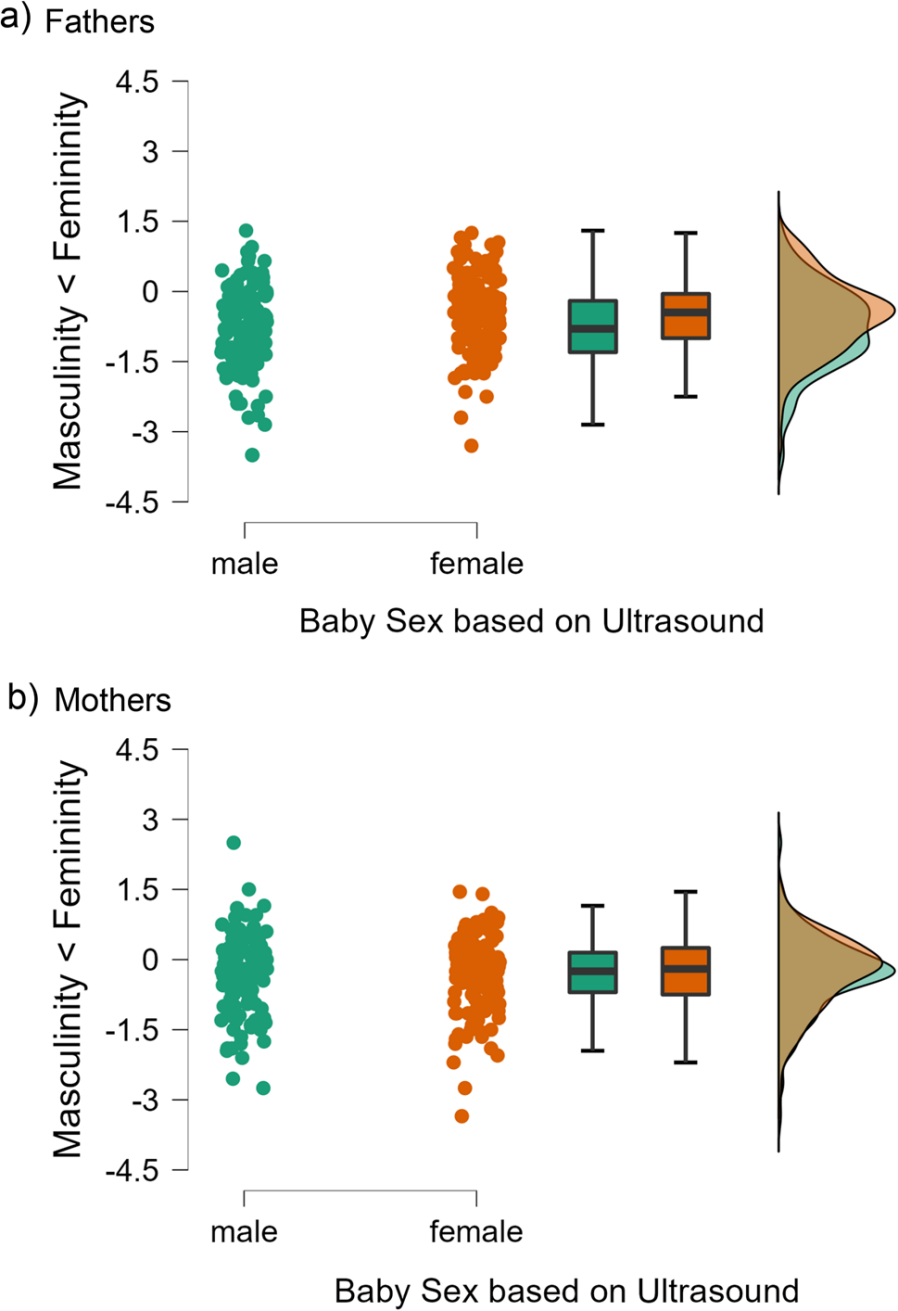
Raincloud plots for desired attributes for their baby on BSRI femininity and masculinity scales (differences score displayed) at T2 as a function of ultrasound sex diagnostic for father (**a**) and mothers (**b**)

This analysis did not yield the expected measurement time by baby sex interaction on the difference score, *F*(1, 207) = 0.02, *p* = 0.903, *η*_*p*_^2^ = 0.00. Instead, this effect was qualified by parent role, *F*(1, 207) = 4.21, *p* = 0.041, *η*_*p*_^2^ = 0.02. Follow-up analyses revealed that fathers overall desired masculine traits more than feminine traits, but more so if ultrasound had suggested they were expecting a boy rather than a girl, *t*(259) = 2.93, *p* = 0.004, *d* = 0.36 (Fig. 2a), whereas mothers’ desires were not contingent on the ultrasound results, *t*(261) = 0.28, *p* = 0.780, *d* = 0.03 (Fig. 2b). These results thus suggest a slight tendency to develop gendered expectations for fathers, but not mothers.

To decompose this finding, we explored which items drove this effect. Using a cutoff criterion of Hedges’ *g* ≥ 0.20 in the expected direction, the item-wise analysis suggested that fathers expecting boys (based on ultrasound) wished their babies to be more daunting, Hedges’ *g* = 0.32, determined, Hedges’ *g* = 0.32, forceful, Hedges’ *g* = 0.23, intelligent, Hedges’ *g* = 0.23, businesslike, Hedges’ *g* = 0.22, and having leadership abilities, Hedges’ *g* = 0.20. Likewise, they wished their children to be less tender, Hedges’ *g* =  − 0.28, sensitive, Hedges’ *g* =  − 0.25, and yielding, Hedges’ *g* =  − 0.23, than fathers expecting girls.

### Do Parents Perceive Their Newborn to Have More Feminine and Masculine Traits, Respectively, Depending on Their Birth Sex?

**Fig. 3:**
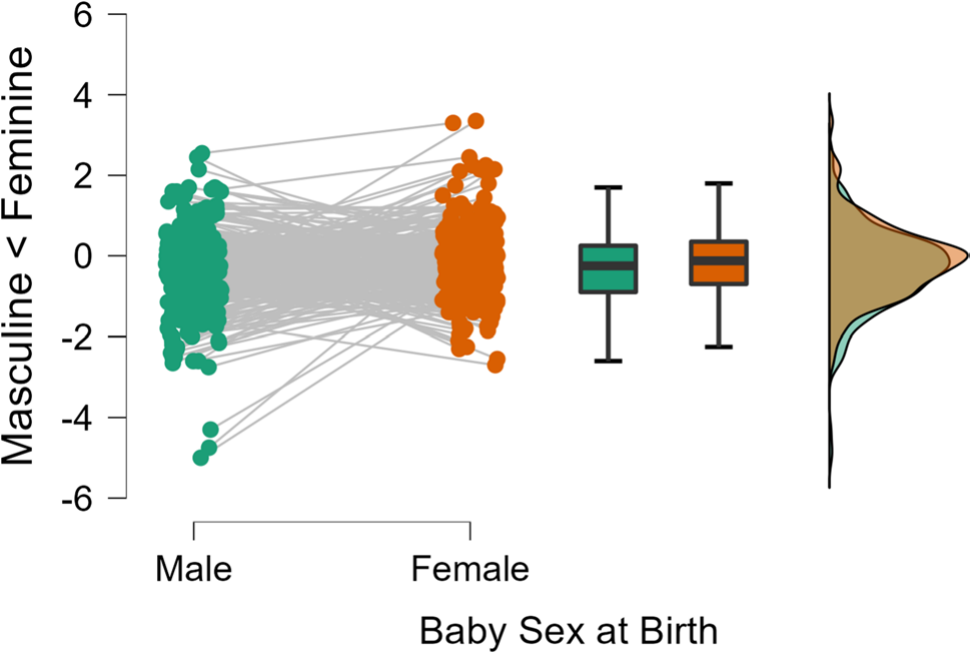
Raincloud plot for perceived baby attributes on BSRI femininity and masculinity scales (differences score displayed) at T3 as a function of sex at birth

Roughly eight weeks after birth parents again completed the BSRI, this time not asking about desired traits but their impression of the baby’s actual traits now that they had got a chance to meet them. To do so, we again subjected the difference scores (dominance of feminine over masculine traits) to a 2 (baby sex; between: boy vs. girl) by 2 (parent; within: mother vs. father) mixed ANOVA. This analysis yielded two main effects. Fathers perceived their babies to have more typically masculine traits than mothers did (independent of the baby’s sex), *F*(1, 284) = 17.56, *p* < 0.001, *η*_*p*_^2^ = 0.06, and newborn boys were perceived to hold more masculine, resp. less feminine traits than newborn girls, *F*(1, 284) = 6.64, *p* = 0.010, *η*_*p*_^2^ = 0.02. There was no significant interaction of these two factors, *F*(1, 284) = 1.51, *p* = 0.167, *η*_*p*_^2^ = 0.01, thus providing no evidence for the notion that mothers and fathers differed in their extent of gendered perception. These findings thus supported the hypothesis of gendered perceptions although the differences were subtle (Fig. 3). Despite the effects for father and mothers not being reliably different from each other, a follow-up inspection suggested that it was reliably different from zero only for fathers, *t*(284) = 2.98, *p* = 0.003, Cohen’s *d* = 0.35, but not for mothers, *t*(285) = 1.06, *p* = 0.292, Cohen’s *d* = 0.12.

A closer inspection of Fig. 3 allows the suspicion that the significant main effect of baby sex was largely driven by three data points for which parents perceived their newborn boy to have a particular dominance of masculine attributes. We thus conducted control analyses without these data points by eliminating all difference scores more extreme than 4. The identical mixed ANOVA still yielded the same results, despite a less pronounced effect of baby sex, *F*(1, 281) = 3.82, *p* = 0.042, *η*_*p*_^2^ = 0.02.

To preclude the possibility that parents’ perceptions are not rooted in stereotypes but their perception of the newborns’ behavior, we added estimations of baby temperament as covariates. Overall, EMA-based temperament judgments showed not perfect but substantial correspondence between mother and father, *r* = 0.546, *p* < 0.001, but neither of them differed as a function of baby sex, *ps* > 0.868. Adding these two EMA-based temperament estimates as covariates yielded the main effect of baby sex intact, *F*(1, 279) = 7.54, *p* = 0.009, *η*_*p*_^2^ = 0.03, even if the three outliers were excluded, *F*(1, 276) = 4.51, *p* = 0.035, *η*_*p*_^2^ = 0.02. These analyses thus lend no support to the suspicion that gendered perceptions of the newborns were due to temperamental differences or the perception thereof.

## Discussion

The present paper presents unique data of parents’ prenatal gendered expectations of their children’s traits. The results revealed relatively small effects of baby sex on gendered expectations, and only for fathers. Once the child was born, both mothers and fathers perceived their child’s personality in sex role consistent ways, albeit again with a small effect size. The modest size of effect is likely not due to the fact that the BSRI scales failed to tap into current sex roles (see also Holt & Ellis, 1998), as fathers and mothers differed markedly in their self-ascription of these traits.

For both, the gendered expectations based on ultrasound diagnostics as well as the gendered perception of newborns, fathers showed more pronounced patterns than mothers. This finding adds further support for the repeated finding that fathers hold more traditional sex stereotypes (Blakemore & Hill, 2008; Endendijk et al., 2013; Tenenbaum & Leaper, 2002), and are more likely to invoke stereotyped norms (Fabes & Martin, 1991). This is particularly relevant as paternal gender socialization, compared with maternal socialization, may be disproportionately influential on child outcomes (Grossmann et al., 2002; Power, 1981). Our study is mute as to why this may be the case. It is conceivable to connect this to men’s greater heteronormative attitudes and less tolerance of gender non-conformity (Duncan et al., 2019) as well as their experienced greater pressure to conform to gender norms (in their case masculinity; Vandello et al., 2008). It should be noted, however, that neither men’s nor women’s benevolent sexism (Glick et al., 2000; which is akin to traditional gender role endorsement) moderated the effect (data available at https://osf.io/crja6).

Despite this consistent pattern, it should also be noted that overall, the effects of baby sex on both expected and perceived traits were comparatively small (Cohen’s *d*’s around 0.36 for both effects as expressed by fathers), also compared to parents’ self-stereotyping on these same scales (with Cohen’s *d*_*z*_’s 0.45 to 0.57). The present data are mute as to whether these substantially larger self-stereotypes are an effect of lifelong gendered socializing, an effect of the cohorts or even just the difference between rating the self vs. others (i.e., one’s baby). It is worth mentioning; however, that many models of sex typing and gender socialization would presume exactly such subtle differences to lead to a self-reinforcing cycle of expectations, perceptions, and self-fulfilling prophecies (Lytton & Romney, 1991). Future research may thus help further tearing these aspects apart.

While the observed effects were small, they provide a unique insight into sex-typing expectations that cannot be well explained by the different temperament and affordances boys and girls bring with them. More recent studies on the perception of children’s physical characteristics typically fail to provide strong effects of sex-consistent stereotyping, but instead show actual physical differences between baby girls and boys can explain differences in parental impressions (Thielmann et al., 2015). We controlled for such associations between behavioral ratings and sex role stereotypes, but found no associations. Likewise, the methodologically strongest papers in this research domain involving so-called gender-labelling studies (babies of either sex clothed in a neutral way and randomly labelled as either a boy or a girl) fail to provide strong evidence for gender stereotyping by adults (Stern & Karraker, 1989). Notably, though, children labelled as girls were regarded as more feminine and less masculine than children labelled as boys (Burnham & Harris, 1992), characterized by others as a mere manipulation check of gender labelling (Thielmann et al., 2015). Our current study not only makes the influence of actual sex differences between boy and girl babies an unlikely candidate, but also show that differential perceptions of femininity and masculinity go far beyond these labels but generalize to relatively broad constructs of masculinity and femininity, respectively.

This of course does not mean that our data speak to the unidirectional role of parent’s sex typing based on their expectations. It does, however, suggest that as much as babies are no blank pages, neither are parents who—once knowing their child’s sex—will form expectations about certain personality traits their child might have. Whether these expectations will then guide parental behavior in a measurable way and ultimately contribute to sex-typing socialization influences (as theorized in the introduction) is beyond the scope of the present paper (for a recent effort at integrating biological, social, and cognitive factors, see Endendijk et al., 2018). Nevertheless, our results show the basic precondition that sex role stereotypes do color parental expectations, even before birth. This is important as there exists an increased recognition that preventing damaging gender attitudes and stereotypes in childhood is key to social change (King et al., 2021).

Although our study had clear strengths by providing prospective prenatal data from a large sample of not only mothers but dyads of becoming parents, there are also limitations. Our measure of baby temperament showed no sex differences. Our interpretation that the different expectations and perception of baby girls and boys is thus not a reaction to actual differences might be premature if these measures simply did not tap into reliable differences that were present nevertheless. One aspect in our data, however, is difficult to reconcile with this view of parental expectations being merely shaped by actual differences. The fact that sex role consistent expectations were more pronounced for fathers than for mothers seems difficult to explain under this perspective. If anything, one would expect that mothers should be in a better position to notice if there were prenatal sex differences (even if they have not-better-than-chance accuracy of predicting gender from fetal movements; Genuis et al., 1996; McFadzen et al., 2017). As the most obvious limitation, however, we present data from one sample in one cultural context. Data from other contexts might provide stronger (or even weaker) evidence for gendered expectations as well as more symmetric effects for mothers and fathers, as there are meaningful cultural differences in gender stereotypes (Nosek et al., 2009). What our data then suggest is that it is a—hitherto largely neglected—possibility that ultrasound information might bias parents’ expectations and perceptions.

In conclusion, it remains a fascinating topic how societal sex roles enter the lives of children and adults and influence their behavior. Our research has elucidated a hitherto largely unexplored piece of the puzzle by showing (admittedly weak) effect of baby sex on gendered expectations even months before the baby was born.

## Data Availability

A complete list of collected variables as well as partial data are openly available at https://osf.io/crja6. Due to the sensitive nature of the data, we asked for participants’ explicit consent to share data publicly. For 261 couples, both partners agreed and only their data are publicly available. Identical analyses on this reduced dataset yield differences in value estimates but not in inferences one would draw from them.
